# Sex-specific differences in bone mineral density loss after sleeve gastrectomy

**DOI:** 10.3389/fmed.2022.957478

**Published:** 2022-10-26

**Authors:** Di Yang, Yafen Ye, Yinfang Tu, Rongrong Xu, Yunfeng Xiao, Hongwei Zhang, Weijie Liu, Pin Zhang, Haoyong Yu, Yuqian Bao, Junfeng Han

**Affiliations:** ^1^Shanghai Clinical Center for Diabetes, Shanghai Diabetes Institute, Shanghai Key Laboratory of Diabetes Mellitus, Department of Endocrinology and Metabolism, Shanghai Jiao Tong University Affiliated Sixth People’s Hospital, Shanghai, China; ^2^Department of Radiology, Shanghai Jiao Tong University Affiliated Sixth People’s Hospital, Shanghai, China; ^3^Department of General Surgery, Shanghai Jiao Tong University Affiliated Sixth People’s Hospital, Shanghai, China

**Keywords:** sex heterogeneity, bone mineral density, sleeve gastrectomy, sex hormone-binding globulin, obesity

## Abstract

**Background:**

Sleeve gastrectomy is an effective bariatric procedure; however, sleeve gastrectomy-related adverse skeletal outcomes have been increasingly reported. High levels of sex hormone-binding globulin (SHBG) have been documented to be a risk factor of bone mineral density (BMD) loss with different effects observed between sexes. The aim of this study was to identify sex-specific changes in BMD following sleeve gastrectomy and to evaluate the role of SHBG in this process.

**Methods:**

This retrospective study included 19 middle-aged men and 30 non-menopausal women with obesity who underwent sleeve gastrectomy in China. Anthropometrics, bone turnover markers, calciotropic hormones, BMD, SHBG, and gonadal steroids were measured preoperatively and at 6 and 12 months postoperatively. Longitudinal changes in BMD, bone turnover markers and SHBG were compared between sexes by linear mixed models. Multiple stepwise regression analysis was used to identify the predictors of BMD loss at the investigated bone sites.

**Results:**

Over the 12-month study period, total hip and femoral neck BMD decreased, while lumbar spine BMD remained largely unchanged in both sexes. Linear mixed models revealed significant sex × time interaction effects in total hip BMD and SHBG, showing that men had a significantly greater reduction in total hip BMD and less increase in SHBG after sleeve gastrectomy than women. In the multivariate model, SHBG was significantly associated with total hip BMD loss in men (adjusted β = −0.533, *P* = 0.019) but not women while total estrogen was significantly associated with total hip BMD loss in women (adjusted β = 0.508, *P* = 0.01) but not men.

**Conclusion:**

Significant sex-specific BMD changes were observed after sleeve gastrectomy in the current study. Sleeve gastrectomy-related increase in SHBG may be a specific risk factor for total hip BMD loss in men. Our results indicate that sex-specific screening may be warranted to facilitate personalized postoperative bone care in this population.

## Introduction

Bariatric surgery (BS) is a common and effective method for treating morbid obesity (1), of which Roux-en-Y gastric bypass and sleeve gastrectomy (SG) (2) are the most commonly performed procedures worldwide. Sleeve gastrectomy (SG) has gradually become the predominant bariatric procedure (2). However, these procedures can adversely affect bone turnover, leading to loss of bone mineral density (BMD) and skeletal fractures (3–5). Many studies have collectively confirmed the detrimental effects of Roux-en-Y gastric bypass on bone mass (6, 7) while the bone remodeling effects of SG are controversial with marked differences between sexes. At 6 months post SG, Wojciech et al. (8) reported significantly decreased BMD at the femoral neck (FN), total hip (TH), and lumbar spine (LS) in 29 obese women, while Adamczyk et al. (9) observed unchanged total body BMD, decreased FN BMD, and increased LS BMD in 25 obese men. Most of the current literature exploring the features of BS-related bone metabolism involved either men or women, with mixed studies including men and women, regardless of menstrual status. Thus, reports on sex-based differences in bone outcomes are scarce.

The cause of sex-based differences of BMD is thought to be multifactorial, including the particular sex hormones and related factors, other hormones regulating the hypothalamic-pituitary-gonadal axis, as well as genetic and mechanical factors. Sex hormones play a crucial role in skeletal integrity. Following SG, previous studies have indicated markable changes in sex hormones leading to the improvement of polycystic ovary syndrome and infertility in women (10, 11) and hypogonadism in men (12, 13) with a general trend toward sex hormones becoming more balanced. In addition, studies have shown that SG causes significant sex-specific changes in sex hormones. In men, an increase in total testosterone (TT) and sex hormone-binding globulin (SHBG) levels, and conversely, unchanged estradiol (E_2_) levels have been observed (13). In women, BS has been shown to significantly increase SHBG levels and decrease total E_2_ and TT levels (14).

SHBG is a liver-derived glycoprotein that binds tightly to testosterone and E_2_ and regulates their bioavailability in the serum (15). Levels of SHBG were reported to be stable during the menstrual cycle (16, 17). Clinically, it has been indicated as a potential biomarker for insulin resistance (18), polycystic ovary syndrome (19) and liver disease (20). Some studies have shown that a higher level of SHBG is a risk factor for osteoporosis and fractures, independent of sex steroid levels (21–24). As SHBG levels have been shown to increase after SG, it is worthwhile to assess whether high level of SHBG can lead to detrimental effects, such as BMD loss, postoperatively.

Few studies have described the role of SHBG in BS-related BMD loss and whether there is a sex discrepancy in the association. Therefore, the main aim of this study was to investigate sex differences in BMD and bone turnover markers (BTMs) at three different bone sites 12 months post SG. The secondary aim was to preliminarily propose the potential association between SHBG levels and SG-related BMD loss.

## Materials and methods

### Study design and participants

In this retrospective study, we recruited a total of 19 men and 30 women with obesity at Shanghai Jiao Tong University Affiliated Sixth People’s Hospital between March 2015 and July 2020. The inclusion criteria were as follows: (1) men aged < 50 years and women aged < 45 years with regular menstrual cycles, and (2) fulfillment of the Chinese SG guideline criteria (25). The exclusion criteria were as follows: (1) usage of medications affecting bone metabolism, including bisphosphonates, teriparatide, and oral glucocorticoids; (2) bone diseases of different etiologies (primary hyperparathyroidism, Paget disease); (3) usage of sex steroid products; (4) missing data on age, sex, body mass index (BMI), BMD, or laboratory investigations; and (5) clinical or laboratory evidence of hepatic or renal disease, gestation or lactation, or malignant disease.

All clinical measurements were recorded at baseline, 6 and 12 months postoperatively. The patients were administered with 50 μg/day of vitamin D3 and 600 mg/day of calcium postoperatively. This study was approved by the Ethics Committee for Human Investigation of Shanghai Jiao Tong University Affiliated Sixth People’s Hospital. Informed consent was obtained from all participants prior to their participation in the study. All methods were performed in accordance with the Declaration of Helsinki of the World Medical Association.

### Laparoscopic sleeve gastrectomy technique

For laparoscopic SG, a 27-French oral gastric tube was placed through the pylorus, near the lesser curvature. Gastric tubulization was performed starting 2–6 cm from the pylorus by resecting straight toward the angle of His. Most of the greater curvature including the complete fundus was removed, with the antrum partially retained, leaving a small gastric tubular pouch of approximately 60–80 mL in volume. Two general surgery specialists performed the SG operations (26) for all patients using standardized laparoscopic techniques.

### Bone mineral density measurement

Dual-energy X-ray absorptiometry [Hologic QDR-2000 (Hologic Corporation, Waltham, MA, United States)] was used to measure areal BMD (g/cm^2^) of the TH, FN, and LS (LS1–4) preoperatively and at 6 and 12 months postoperatively. The coefficient of variation was 0.8–1.0% for areal BMD. Prodigy Encore software (version 6.70, standard-array mode; GE Healthcare) was used for image analysis, as previously described (27). Two medically trained technicians performed the dual-energy X-ray absorptiometry scans and analyzed the images.

### Clinical and laboratory assessments

Anthropometric and laboratory parameters, including sex hormone levels, bone metabolic indexes, and glucose-lipid profiles, were assessed preoperatively and 6 and 12 months postoperatively. Anthropometric measurements, including height (cm), weight (kg), and waist circumference (cm), were recorded using standardized methods (27). BMI (kg/m^2^) was calculated as weight (kg)/[height (m)]^2^. The excess weight loss percentage was calculated as (preoperative weight - current weight)/(preoperative weight - ideal weight) where ideal weight was based on a BMI of 24 kg/m^2^ (28). The percentage of total weight loss was calculated as 100 × (initial weight–postoperative weight)/initial weight. Laboratory parameters were determined using overnight fasting venous blood samples. Sex hormones, including total serum SHBG, TT, and total E_2_, were measured using an automated Roche electrochemiluminescence system (Roche Diagnostics GmbH, Germany). The bone metabolism variables of parathyroid hormone, 25-hydroxy vitamin D, procollagen type I amino-terminal propeptide (P1NP), N-terminal osteocalcin (N-MID), and β-cross-linked C-telopeptide of type I collagen (β-CTX), were determined by Roche electrochemiluminescence system (Roche Diagnostics GmbH, Germany). Plasma glucose levels were determined using the glucose oxidase method and glycated hemoglobin levels were measured using high-performance liquid chromatography (Bio-Rad Inc., Hercules, CA, United States). Serum insulin was assayed using radioimmunoassay with specific insulin detection kits (Roche Diagnostics [Shanghai] Limited Company, Shanghai, China). Homeostatic model assessment for insulin resistance was calculated as fasting insulin (mIU/L) × fasting glucose (mM)/22.5. The homeostasis model assessment of β-cell function was assessed as [20 × insulin (mIU/mL)]/[glucose (mmol/L)– 3.5] (29). Lipid profiles including serum total cholesterol, total triglycerides, high-density lipoprotein cholesterol, and low-density lipoprotein cholesterol levels were analyzed using standard enzymatic methods with a 7600–120 Hitachi automatic analyzer (Hitachi, Tokyo, Japan).

### Statistical analyses

Data distribution was evaluated using the Shapiro–Wilk test. Normally distributed data were reported as mean ± standard deviation, while skewed data were reported as median (interquartile range). The skewed variables were logarithmically transformed (log-transformed) before the analysis. The clinical characteristics at 6 or 12 months after SG were compared with baseline using a paired-samples test or Wilcoxon signed-rank test within each sex group. A linear mixed effects model was used to assess the time and sex effects of bone metabolic variables and SHBG. The estimated marginal means of BTMs and SHBG were derived from the log-transformed data. After adjusting for baseline values, the percent change from baseline to 6 or 12 months postoperatively was calculated as 100 × (initial measurement–postoperative measurement)/initial measurement. Correlations between TH BMD and clinical variables including SHBG and sex steroids were assessed using single linear regression analysis, while potential variables accounting for BMD were entered into multiple stepwise regression analysis. These analyses were performed as subgroup analyses by sex to determine between-group differences. Significance was set at a two-sided test with a *P* value < 0.05. All statistical analyses were performed using the IBM Statistical Product and Service Solutions Statistics for Windows (version 26.0; IBM Corp., Armonk, NY, United States).

## Results

### Sleeve gastrectomy-related changes in anthropometrics and metabolic characteristics

This retrospective study included 49 participants with a mean age of 32.9 ± 7.80 years with no significant difference between sexes. The comparable mean BMI at baseline was 35.01 ± 4.71 kg/m^2^ *(P* > 0.05). The mean waist circumference and waist-to-hip ratio at baseline were 110.88 ± 9.82 cm and 0.97 ± 0.06, respectively, with a lower waist-to-hip ratio observed in women. Anthropometric values decreased postoperatively over 12 months in both men and women. TT levels significantly increased in men (11.77 ± 4.31 nM vs. 19.86 ± 6.38 nM, baseline vs. 12 months follow-up, *P* < 0.001), but decreased in women (1.13 ± 0.52 nM vs. 0.73 ± 0.34 nM, baseline vs. 12 months follow-up, *P* < 0.027). Levels of E_2_ significantly increased in women over the 12 months (221.01 ± 155.29 nM vs. 418.052 ± 369.23 nM, *P* < 0.016), but no significant change were observed in men (*P* = 0.298). Except for the total cholesterol and low-density lipoprotein cholesterol levels, the glucose-lipid metabolic variables improved significantly in both sexes, as expected *(P* < 0.05). Serum calcium levels remained stable *(P* > 0.05). 25-hydroxy vitamin D levels significantly increased in women (13.73 ± 5.54 mM vs. 18.92 ± 9.59 mM) but remained largely unchanged in men. Phosphorus levels increased *(P* < 0.05) while parathyroid hormone levels did not change significantly over 12 months *(P* > 0.05) in both sexes. Most of the anthropometric and clinical characteristics of participants varied as a function of SG, and the mean values were shown within each sex group in Table 1 with the distribution of data in Supplementary Table 1.

**TABLE 1:** Clinical characteristics of the participants at baseline and at 6 and 12 months after sleeve gastrectomy.

			Men					Women		
		
	baseline	6 months	*P-value*	12 months	*P-value*	baseline	6 months	*P-value*	12 months	*P-value*
Age, years	34.11 ± 9.01					32.13 ± 6.98				
Weight, kg	107.24 ± 13.21	77.19 ± 10.31	** < 0.001**	78.54 ± 9.37	** < 0.001**	96.35 ± 12.58	71.53 ± 9.76	** < 0.001**	68.32 ± 9.38	** < 0.001**
BMI, kg/m^2^	34.25 ± 3.91	25.05 ± 2.43	** < 0.001**	25.24 ± 2.78	** < 0.001**	35.64 ± 5.12	26.87 ± 4.08	** < 0.001**	25.21 ± 3.62	** < 0.001**
WC, cm	111.36 ± 9.71	90.31 ± 7.51	** < 0.001**	87.09 ± 8.60	** < 0.001**	110.62 ± 10.30	88.42 ± 10.18	** < 0.001**	84.50 ± 8.44	** < 0.001**
WHR	0.99 ± 0.05	0.93 ± 0.06	** < 0.001**	0.90 ± 0.07	** < 0.001**	0.95 ± 0.06	0.89 ± 0.07	** < 0.001**	0.89 ± 0.06	** < 0.001**
SHBG, nM	15.9 (10.6, 21.9)	36.7 (20.75, 45.15)	**0.001**	36.59 ± 15.99	** < 0.001**	21.62 ± 8.65	55.86 ± 27.21	** < 0.001**	61.70 ± 33.03	** < 0.001**
TT, nM	11.77 ± 4.31	20.29 ± 6.82	**0.005**	18.6 (14.4, 27.2)	** < 0.001**	1.13 ± 0.52	0.74 ± 0.33	**0.047**	0.73 ± 0.34	**0.027**
E2, pM	158.89 ± 35.42	152.25 ± 42.71	0.56	150.67 ± 30.76	0.298	157 (111.75, 303.75)	328.48 ± 179.93	0.209	312.5 (156.75, 425.00)	**0.012**
TC, μM	4.37 ± 1.08	4.47 ± 1.16	0.61	4.25 ± 0.93	0.657	4.71 ± 0.79	4.86 ± 0.89	0.601	4.54 ± 0.61	0.287
TG, mM	2.20 ± 1.33	0.97 ± 0.31	**0.003**	0.91 ± 0.47	** < 0.001**	1.68 ± 0.67	1.01 ± 0.31	** < 0.001**	0.84 ± 0.39	** < 0.001**
HDL-C, mM	0.92 ± 0.19	1.31 ± 0.25	** < 0.001**	1.27 ± 0.26	** < 0.001**	1.05 ± 0.21	1.12 ± 0.18	0.05	1.39 ± 0.21	** < 0.001**
LDL-C, mM	2.68 ± 0.93	2.62 ± 0.93	0.428	2.44 ± 0.78	0.138	2.88 ± 0.77	2.94 ± 0.75	0.761	2.57 ± 0.57	0.151
FPG, mM	7.52 ± 2.78	4.98 ± 0.82	**0.001**	4.89 ± 0.74	** < 0.001**	5.39 (4.86, 7.38)	5.14 ± 1.23	** < 0.001**	4.83 ± 1.03	**0.001**
HbA1c,%	7.35 ± 1.70	5.50 ± 0.46	** < 0.001**	5.53 ± 0.40	** < 0.001**	6.50 ± 1.96	5.49 ± 0.86	**0.032**	5.46 ± 0.63	**0.02**
FINS, Mu/L	28.61 (13.46, 35.9)	7.82 ± 5.55	**0.003**	7.61 ± 3.82	** < 0.001**	26.11 ± 17.01	7.13 (5.29, 8.81)	** < 0.001**	7.26 ± 4.01	** < 0.001**
HOMA-IR	8.61 (5.62, 11.53)	1.81 ± 1.42	**0.002**	1.81 ± 1.01	** < 0.001**	7.99 ± 8.27	1.44 (1.09, 2.18)	** < 0.001**	1.32 ± 1.17	** < 0.001**
Calcium, mM	2.28 ± 0.10	2.35 ± 0.10	**0.011**	2.28 ± 0.08	0.876	2.23 ± 0.08	2.29 ± 0.09	0.052	2.21 ± 0.12	0.272
Phosphorus, mM	1.29 ± 0.12	1.34 ± 0.18	0.396	1.39 ± 0.20	**0.034**	1.24 ± 0.20	1.34 ± 0.18	0.116	1.34 ± 0.14	**0.011**
25(OH)D, mM	14.90 ± 4.95	20.87 (16.10, 24.93)	**0.008**	17.15 ± 4.93	0.106	13.73 ± 5.54	21.48 ± 8.53	**0.001**	18.92 ± 9.59	**0.011**
PTH, pM	42.46 ± 13.68	36.35 ± 16.89	0.329	41.76 ± 14.56	0.868	44.07 ± 14.04	39.86 ± 18.36	0.353	40.20 ± 16.45	0.52

Data are presented as the mean ± standard deviation or median (Q25, Q75).

BMI, body mass index; WC, waist circumference; WHR, waist-to-hip ratio; SHBG, sex hormone–binding globulin; TT, total testosterone; E_2_, estrodial; TC, total cholesterol; TG, total triglycerides; HDL-C, high-density lipoprotein cholesterol; LDL-C, low-density lipoprotein cholesterol; FPG, fasting plasma glucose; HbA1c, glycated hemoglobin; FINS, fasting insulin; HOMA-IR, homeostasis model assessment of insulin resistance; 25(OH)D, serum 25-hydroxyvitamin D; PTH, serum parathyroid hormone. *P* values (bold) indicate statistical significance compared with baseline in each sex group.

### Sex discrepancy of sleeve gastrectomy-related changes in bone mineral density

**FIGURE 1:**
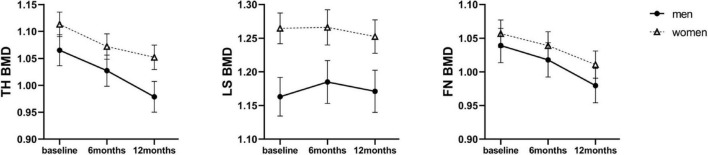
Estimated marginal means (y-axis) of BMD for men and women group over time points (x-axis). Error bars indicate standard error. BMD, bone mineral density; TH, total hip; LS, lumbar spine; FN, femoral neck.

During the 12-month follow-up period, none of the patients developed osteoporosis. The mean BMD values at three sites at baseline and 6 and 12 months after SG were shown in Supplementary Table 2. TH BMD and FN BMD was comparable between sexes at baseline, respectively. The mean LS BMD level was higher in women than in men at baseline (1.253 ± 0.135 g/cm2 vs. 1.166 ± 0.103 g/cm2, *P* < 0.01). Longitudinal data analysis, according to linear mixed effects models, showed a significant SG-related impact of BMD loss at TH (*P* < 0.001) and FN (*P* < 0.001) over 12 months, but not at LS (*P* = 0.234) in both sexes. The dynamic changes of BMDs across different time points in men and women were present in Figure 1, with estimated marginal means and corresponding standard errors. When investigating the interaction effect of sex and SG across time points, men demonstrated greater TH BMD loss compared to women at 12 months (β = 0.025, SE = 0.009, *P* = 0.008) (Table 2). Overall, men with obesity might be more vulnerable to SG-related BMD loss than women with obesity, especially for TH BMD.

**TABLE 2:** Linear mixed effects in sex- and sleeve gastrectomy -related changes of bone metabolic variables and SHBG at 6 and 12 months of follow-up.

	TH BMD	LS BMD	FN BMD	P1NP	N-MID	β -CTX	SHBG
Sex^†^	−0.048 (0.037)	−0.102 (0.037)**	−0.018 (0.033)	0.10 (0.11)	0.16 (0.10)	0.38 (0.13) **	−0.26 (0.15)
TP1-TP2	−0.041 (0.008)**	0.001 (0.012)	−0.018 (0.007)**	0.22 (0.09) *	0.45 (0.07) **	0.93 (0.10) **	0.91 (0.07)**
TP1-TP3	−0.061 (0.006) **	−0.012 (0.010)	−0.046 (0.007)**	0.23 (0.06) **	0.43 (0.06) **	0.73 (0.07) **	0.92 (0.06)**
sex × TP1-TP2	0.003 (0.012)	0.020 (0.018)	−0.004 (0.010)	0.34 (0.13) *	0.28 (0.11) *	0.09 (0.14)	−0.12 (0.11)*
sex × TP1-TP3	−0.025 (0.009)**	0.020 (0.016)	−0.014 (0.011)	0.28 (0.10) **	0.33 (0.09) **	0.11 (0.11)	−0.27 (0.09)**
Constant	1.113 (0.023)**	1.265 (0.023)**	1.057 (0.020)**	3.89 (0.07) **	2.54 (0.06) **	5.64 (0.08) **	3.06 (0.09)**

Values indicate the estimated effect (β) and corresponding standard error (SE). TP; Time point; BMD, bone mineral density; FN, femoral neck; TH, total hip; LS, lumbar spine; P1NP, procollagen type I amino-terminal propeptide; N-MID, N-terminal osteocalcin; β-CTX, β-cross-linked C-telopeptide of type I collagen; SHBG, sex hormone–binding globulin. TP1 = baseline; TP2 = 6 months after sleeve gastrectomy; TP3 = 12 months after sleeve gastrectomy.

^†^Sex 1 for men and 2 for women.

*Indicates *p* < 0.05.

**Indicates *p* < 0.01.

### Sex discrepancy of sleeve gastrectomy-related changes in bone turnover markers

**FIGURE 2:**
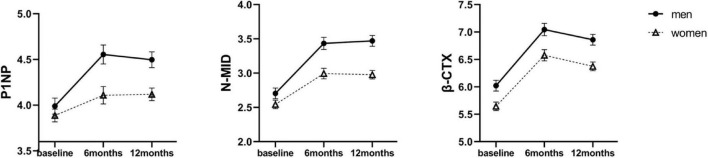
Estimated marginal means (y-axis) of bone turnover markers for men and women group over time points (x-axis). Error bars indicate standard error. P1NP, procollagen type I amino-terminal propeptide; β-CTX, β-cross-linked C-telopeptide of type I collagen; N-MID, N-terminal osteocalcin.

Bone tissue undergoes continuous remodeling to maintain a dynamic balance between bone formation and resorption, which contributes to BMD. The following serum markers of bone remodeling were measured: P1NP and N-MID, representing bone formation, and β-CTX representing bone resorption. The raw mean values of BTMs at baseline and 6 and 12 months after SG are shown in Supplementary Table 2. The level of β-CTX was higher in men than in women at baseline (463.09 ± 181.59 mg/mL vs. 322.57 ± 182.10 mg/mL, *P* < 0.05). All values of BTMs increased in both sexes across 12 months (Figure 2). When investigating the different effects of SG between sexes across time points, linear mixed effects models revealed higher levels of P1NP and N-MID in men than women at both 6 and 12 months (Table 2). Therefore, the bone activity was greater in men than that in women 12 months after SG.

### Sex discrepancy of sleeve gastrectomy-related changes in serum sex hormone-binding globulin

**FIGURE 3:**
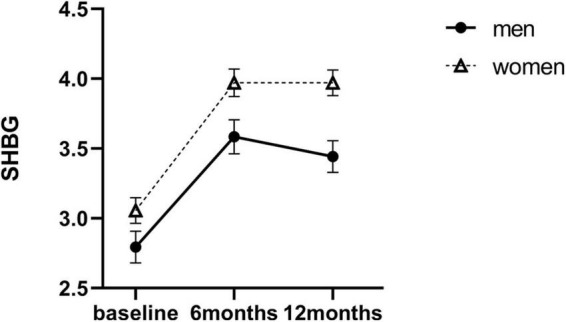
Estimated marginal means (y-axis) of SHBG for men and women group over time points (x-axis). Error bars indicate standard error. SHBG, sex hormone–binding globulin.

There was no significant sex difference in SHBG levels at baseline [18.42 ± 7.93 nM (men) vs. 21.62 ± 8.65 nM (women), *P* = 0.144]. Compared with baseline, SHBG levels increased to 38.59 ± 17.24 nM at 6 months and decreased slightly to 36.59 ± 15.99 nM at 12 months in men (*P* < 0.001). In women, SHBG levels also increased progressively over the 12 months to 61.70 ± 33.03 nM (*P* < 0.001). The linear mixed model shown significant main interaction effects of sex × time for SHBG at 6 months (β = −0.12, SE = 0.11, *P* = 0.019) and 12 months (β = −0.27, SE = 0.09, *P* = 0.001) after SG. This indicated that the increase in SHBG levels in men was far less than that in women over the 12 months (Table 2 and Figure 3).

### Potential variables accounting for total hip bone mineral density loss between sexes

To evaluate potential variables related to the sex-specific TH BMD loss, single linear regression analysis was performed in both sexes (Table 3). Subsequently, stepwise multiple logistic regression analysis was performed to identify potential independent variables of TH BMD loss. The results of these analyses are presented for men and women separately in Table 4.

**TABLE 3:** Correlation analysis between percent change between clinical and bone metabolic variables.

	Men		Women	
	TH BMD		TH BMD	
% change	β	*P-value*	β	*P-value*
SHBG	−0.533	**0.019**	−0.117	0.546
TT	−0.433	0.064	−0.035	0.876
E_2_	0.166	0.51	0.564	**0.008**
EWL%	−0.097	0.702	0.306	0.113
TWL%	0.351	0.14	0.165	0.403
WHR	0.275	0.27	0.061	0.763
TC	−0.001	0.996	−0.084	0.718
TG	−0.262	0.279	−0.181	0.434
HDL-C	0.177	0.482	0.056	0.809
LDL-C	0.177	0.482	−0.016	0.945
FPG	−0.073	0.766	−0.284	0.212
HbA1c	−0.299	0.214	0.096	0.68
FINS	−0.031	0.902	−0.576	**0.01**
HOMA-IR	0.132	0.59	−0.615	**0.005**
PTH	0.308	0.214	−0.39	0.089
25(OH)D	0.303	0.272	−0.205	0.36
Calcium	−0.133	0.586	0.133	0.576
P1NP	−0.182	0.455	0.162	0.402
β-CTX	0.12	0.634	−0.088	0.651
N-MID	−0.195	0.425	0.169	0.382

SHBG, sex hormone–binding globulin; TT, total testosterone; E_2_, estradiol; EWL%, excess weight loss percentage; TWL%, total weight loss; WHR, waist-to-hip ratio; TC, total cholesterol; TG, total triglycerides; HDL-C, high-density lipoprotein cholesterol; LDL-C, low-density lipoprotein cholesterol; FPG, fasting plasma glucose; HbA1c, glycated hemoglobin; FINS, fasting insulin; HOMA-IR, homeostasis model assessment of insulin resistance; FN, femoral neck; TH, total hip; LS, lumbar spine; P1NP, procollagen type I amino-terminal propeptide; N-MID, N-terminal osteocalcin; β-CTX, β-cross-linked C-telopeptide of type I collagen; 25(OH)D, serum 25-hydroxyvitamin D; PTH, serum parathyroid hormone. *P* values (bold) indicate statistical significance.

**TABLE 4:** Multiple stepwise regression analysis of potential factors associated with TH BMD loss.

		Men				Women		
		
Dependent variables	Independent variables	Adjusted β	*P*	Adjusted *R*^2^	Independent variables	Adjusted β	*P*	Adjusted *R*^2^
	Δ1% SHBG	−0.533	0.019	0.242	Δ1% HOMA-IR	−0.62	0.003	
Δ% TH BMD								0.657
					Δ% E_2_	0.508	0.01	

Δ% BMD of TH in men adjusted for age and percentage change in SHBG and TT.

Δ% BMD of TH in women adjusted for age, E_2_, FINS, and HOMA-IR.

SHBG, sex hormone-binding globulin; BMD, bone mineral density; TH, total hip; TT, total testosterone; E_2_, estrogen; FINS, fasting insulin; HOMA-IR, homeostasis model assessment of insulin resistance.

For men, SHBG (adjusted β = −0.533, *P* = 0.019) was the best predictor of TH BMD loss (R^2^ = 0.242). For women, the best predictors for TH BMD loss were homeostatic model assessment for insulin resistance (adjusted β = −0.62, *P* = 0.003) and E_2_ (adjusted β = 0.508, *P* = 0.01) (R^2^ = 0.657). This indicated that SHBG might play a key role in TH BMD loss in men, but not in women. In conclusion, predictors for TH BMD loss are different between men and women.

## Discussion

In this longitudinal study, we observed significant sex-related differences in SG-related bone remodeling and in the association between SHBG and BMD. Although BMD was reduced postoperatively in both men and women, men had significantly more TH BMD loss and relatively higher levels of bone formation markers (P1NP and N-MID) than women. Changes in SHBG level was negatively associated with TH BMD loss in men, whereas in women, SHBG levels was not associated with BMD loss at any of the measured sites.

Sex-related differences in BMD changes after SG have not been clearly or extensively described in the literature. A previous meta-analysis (30) reported that TH and FN BMD decreased in both sexes 12 months after SG, but LS BMD remained unchanged. In this regard, our data are largely in agreement with the results. Furthermore, our linear mixed effects models indicated that TH BMD reduction was more profound in men than women, and no significant sex difference of FN BMD levels was observed. In contrast to our results, a study in Romania reported significant BMD loss in men at all investigated sites, including LS and FN, compared with women (31) and a Chinese study conducted by Wang et al. (32) found that BMD was significantly decreased in women but unchanged in men after SG. Variations in study parameters such as ethnicity, age of participants, and study period may explain these differences.

Studies have proved that bone remodeling is activated to a lesser extent after SG than after Roux-en-Y gastric bypass (33, 34). However, sex discrepancies in BTM changes after BS have rarely been reported. In the Chinese population, Wang et al. (32) found that type I collagen and osteocalcin increased in both men and women after SG. In line with this study, our data shown that all BTMs including P1NP, N-MID and β-CTX increased postoperatively. These results indicated that in both men and women, SG enhanced the bone remodeling. Furthermore, we displayed the effects of sex and time in BTM changes after SG in linear mixed effects model, revealing a more significant increase in P1NP and N-MID in men than women. Our results indicated that men were more susceptible to BMD loss, especially TH BMD loss, which may be due to the greater activation of bone turnover in men.

A possible explanation for sex-specific SG-related BMD loss could be the postoperative endocrine-disrupting chemicals. They affect estrogenic or androgenic signaling pathways or act independently of the sex steroid axis. Higher levels of E_2_, as observed in menstruating women, is a widely accepted factor for the sex disparity in BMD. This might explain the positive relationship of percentile change between E_2_ and TH BMD observed in women but not in men in our study. Correspondingly, levels of SHBG, which modulate sex hormones, might also be relevant to the sex-specific BMD changes after SG. A higher SHBG level has been associated with BMD loss (21, 35). Moreover, previous studies have reported that SG causes a significant increase in SHBG levels in men (13) and women (14) separately. Our study found that, over 12 months, SHBG levels increased in both sexes, with a greater effect in women. However, increase in SHBG levels were inversely related to TH BMD loss in men but were not associated with BMD loss at any site in women. Yang et al. (36) conducted a cross-sectional study of 6,434 participants to examine the association between sex hormone levels and BMD and found a differential effect of SHBG on BMD by sex, which was similar to our findings.

The mechanism by which SHBG influences BMD in different sexes remains unclear. One possible explanation for the sex disparity may be the “free hormone hypothesis” (37), whereby SHBG acts as a biologically inert buffer to modulate and transport androgens or estrogens from circulation to bone cells with various affinities to sex steroids: dihydrotestosterone > testosterone > androstenediol > E_2_ > estrone (19). Grasa et al. (38) also reported that the binding capacity of SHBG was affected by sex due to the differences in structure and affinities of gonadal steroids (binding sites are mostly occupied by testosterone due to a lower estrogen-binding capacity in men). In addition to the indirect effect of SHBG on BMD, SHBG can directly participate in signal transduction in bone cells with an SHBG membrane receptor (39) or by endocytic facilitation of SHBG-bound androgens and estrogens (40). Moreover, genetic polymorphisms in *SHBG* might also contribute to the difference in BMD loss between men and women. Haplotype CCGGT (consisting of rs11078701C, rs1017163C, rs9898876G, rs62059836G, and rs2541012T) and haplotype CGGT (consisting of rs858521C, rs858518G, rs6259G, and rs727428T) in *SHBG* and neighboring genes have been associated with a significant risk effect for osteoporosis in Chinese men (41). In addition, polymorphism in the 5’UTR (G to A base change) of rs1799941 has been associated with lower TH BMD in postmenopausal women (42). However, further studies are needed to elucidate the sex-specific genetic variants of SHBG in BMD loss following SG.

To the best of our knowledge, this is the first study to show sex differences in post-SG BMD loss between middle-aged Chinese men and non-menopausal Chinese women by using linear mixed effects model. Furthermore, the detrimental effects of increased SHBG levels on BMD in men who underwent SG were demonstrated for the first time. Our results indicate the need for personalized bone care. However, this study has some limitations. First, the sample size was not large enough to draw definitive conclusions. Second, its retrospective nature may have led to a bias, including the lack of data pertaining to other factors which may affect bone health such as patients’ activity levels and nutritional intake. Third, causality cannot be assumed by this observational study, and this pilot study can only propose the possible link between SHBG levels and SG-related BMD loss. Further studies with larger sample sizes, longer follow-up periods, and polymorphisms in SHBG testing may be needed to evaluate the utility of these results in providing personalized care for patients post SG. In addition, animal or cell experiments are needed to elucidate the exact mechanisms for SHBG on bone metabolism.

In conclusion, men were more susceptible than women to the detrimental effects of SG on BMD, particularly TH BMD. This may be related to increased levels of SHBG after SG. Therefore, early targeted screening and intervention may be necessary for patients undergoing this surgical procedure. Furthermore, the determination of serum SHBG levels may be a useful screening tool for a personalized bone care postoperatively.

## Data availability statement

The original contributions presented in this study are included in the article/Supplementary material, further inquiries can be directed to the corresponding author/s.

## Ethics statement

The studies involving human participants were reviewed and approved by the Ethics Committee for Human Investigation of Shanghai Jiao Tong University Affiliated Sixth People’s Hospital. The patients/participants provided their written informed consent to participate in this study.

## Author contributions

DY, JH, and HY designed the study. DY, YY, and YT analyzed the data, prepared the figures, and drafted the manuscript. RX and YX recruited participants and collected the data. HZ, WL, and PZ were responsible for bariatric surgery. YB, JH, and HY contributed to the discussion and revised the manuscript. All authors have read and approved the final study.

## Abbreviations

BS: bariatric surgery
SG: sleeve gastrectomy
BMD: bone mineral density
FN: femoral neck
TH: total hip
LS: lumbar spine
TT: total testosterone
E_2_: estradiol
SHBG: sex hormone-binding globulin
BTM: bone turnover marker
P1NP: procollagen type I amino-terminal propeptide
N-MID: N-terminal osteocalcin
β -CTX: β -cross-linked C-telopeptide of type I collagen.
