# Supplementary material for: Sex-specific differences in bone mineral density loss after sleeve gastrectomy
Source: Front Med (Lausanne). 2022 Oct 26;9:957478. doi: 10.3389/fmed.2022.957478 (PMC9644159; doi:10.3389/fmed.2022.957478)
Supplement: Supplementary file 1 [file Data_Sheet_1.docx]

**SUPPLEMENTARY TABLE1** Shapiro–Wilk test of clinical characteristics at baseline and at 6 and 12 months after sleeve gastrectomy

| **men** | **baseline** | |  | **6months** | |  | **12months** | |
| --- | --- | --- | --- | --- | --- | --- | --- | --- |
|  | ***W*** | ***P-value*** |  | ***W*** | ***P-value*** |  | ***W*** | ***P-value*** |
| Weight, kg | 0.982 | 0.975 |  | 0.913 | 0.301 |  | 0.963 | 0.823 |
| BMI, kg/m^2^ | 0.904 | 0.243 |  | 0.955 | 0.733 |  | 0.979 | 0.959 |
| WC, cm | 0.916 | 0.328 |  | 0.93 | 0.45 |  | 0.953 | 0.705 |
| WHR | 0.963 | 0.822 |  | 0.902 | 0.142 |  | 0.909 | 0.271 |
| SHBG, nM | 0.822 | **0.013** |  | 0.868 | **0.049** |  | 0.931 | 0.348 |
| TT, nM | 0.954 | 0.655 |  | 0.893 | 0.107 |  | 0.857 | **0.035** |
| E2, pM | 0.935 | 0.398 |  | 0.93 | 0.338 |  | 0.922 | 0.268 |
| TC, μM | 0.842 | 0.078 |  | 0.952 | 0.729 |  | 0.978 | 0.955 |
| TG, mM | 0.875 | 0.169 |  | 0.9 | 0.291 |  | 0.914 | 0.386 |
| HDL-C, mM | 0.89 | 0.236 |  | 0.943 | 0.644 |  | 0.887 | 0.218 |
| LDL-C, mM | 0.955 | 0.765 |  | 0.967 | 0.873 |  | 0.978 | 0.954 |
| FPG, mM | 0.924 | 0.463 |  | 0.875 | 0.17 |  | 0.872 | 0.156 |
| HbA1c, % | 0.852 | 0.1 |  | 0.955 | 0.761 |  | 0.906 | 0.326 |
| FINS, Mu/L | 0.774 | **0.01** |  | 0.852 | 0.079 |  | 0.937 | 0.554 |
| HOMA-IR | 0.806 | **0.024** |  | 0.847 | 0.069 |  | 0.93 | 0.486 |
| Calcium, mM | 0.928 | 0.496 |  | 0.868 | 0.144 |  | 0.952 | 0.726 |
| Phosphorus, mM | 0.889 | 0.23 |  | 0.839 | 0.074 |  | 0.829 | 0.058 |
| 25(OH)D, mM | 0.936 | 0.536 |  | 0.801 | **0.021** |  | 0.926 | 0.44 |
| PTH, pM | 0.977 | 0.945 |  | 0.936 | 0.535 |  | 0.927 | 0.453 |
| TH BMD, g/cm^2^ | 0.916 | 0.222 |  | 0.905 | 0.156 |  | 0.953 | 0.652 |
| LS BMD, g/cm^2^ | 0.985 | 0.995 |  | 0.886 | 0.085 |  | 0.892 | 0.104 |
| FN BMD, g/cm^2^ | 0.962 | 0.786 |  | 0.967 | 0.853 |  | 0.936 | 0.404 |
| P1NP, mg/mL | 0.968 | 0.876 |  | 0.98 | 0.963 |  | 0.927 | 0.422 |
| N-MID, mg/mL | 0.913 | 0.299 |  | 0.941 | 0.564 |  | 0.976 | 0.941 |
| β-CTX, mg/mL | 0.95 | 0.673 |  | 0.856 | 0.068 |  | 0.937 | 0.525 |
| **women** |  |  |  |  |  |  |  |  |
| Weight, kg | 0.954 | 0.74 |  | 0.969 | 0.837 |  | 0.879 | 0.333 |
| BMI, kg/m^2^ | 0.788 | 0.083 |  | 0.938 | 0.645 |  | 0.946 | 0.693 |
| WC, cm | 0.809 | 0.119 |  | 0.789 | 0.084 |  | 0.836 | 0.184 |
| WHR | 0.893 | 0.395 |  | 0.874 | 0.312 |  | 0.781 | 0.072 |
| SHBG, nM | 0.911 | 0.22 |  | 0.921 | 0.291 |  | 0.903 | 0.176 |
| TT, nM | 0.916 | 0.252 |  | 0.948 | 0.601 |  | 0.937 | 0.457 |
| E2, pM | 0.846 | **0.032** |  | 0.969 | 0.895 |  | 0.811 | **0.012** |
| TC, μM | 0.919 | 0.531 |  | 0.856 | 0.246 |  | 0.906 | 0.462 |
| TG, mM | 0.967 | 0.491 |  | 0.981 | 0.909 |  | 0.941 | 0.663 |
| HDL-C, mM | 0.994 | 0.979 |  | 0.917 | 0.519 |  | 0.864 | 0.274 |
| LDL-C, mM | 0.813 | 0.127 |  | 0.841 | 0.198 |  | 0.78 | 0.071 |
| FPG, mM | 0.699 | **0.011** |  | 0.863 | 0.272 |  | 0.998 | 0.993 |
| HbA1c, % | 0.975 | 0.874 |  | 0.801 | 0.103 |  | 0.773 | 0.062 |
| FINS, Mu/L | 0.897 | 0.393 |  | 0.672 | **0.005** |  | 0.966 | 0.851 |
| HOMA-IR | 0.925 | 0.566 |  | 0.688 | **0.007** |  | 0.983 | 0.951 |
| Calcium, mM | 0.863 | 0.272 |  | 0.973 | 0.861 |  | 0.946 | 0.689 |
| Phosphorus, mM | 0.768 | 0.056 |  | 0.975 | 0.875 |  | 0.964 | 0.803 |
| 25(OH)D, mM | 0.82 | 0.117 |  | 0.905 | 0.436 |  | 0.869 | 0.261 |
| PTH, pM | 0.803 | 0.086 |  | 0.798 | 0.079 |  | 0.982 | 0.945 |
| TH BMD, g/cm^2^ | 0.952 | 0.666 |  | 0.928 | 0.389 |  | 0.898 | 0.173 |
| LS BMD, g/cm^2^ | 0.937 | 0.487 |  | 0.907 | 0.225 |  | 0.958 | 0.746 |
| FN BMD, g/cm^2^ | 0.965 | 0.836 |  | 0.95 | 0.638 |  | 0.951 | 0.658 |
| P1NP, mg/mL | 0.817 | **0.015** |  | 0.78 | **0.006** |  | 0.808 | **0.012** |
| N-MID, mg/mL | 0.835 | **0.024** |  | 0.831 | **0.021** |  | 0.865 | 0.057 |
| β-CTX, mg/mL | 0.813 | **0.013** |  | 0.893 | 0.129 |  | 0.863 | 0.053 |

**Abbreviations:** BMI, body mass index; WC, waist circumference; WHR, waist-to-hip ratio; SHBG, sex hormone–binding globulin; TT, total testosterone; E_2_,estrodial; TC, total cholesterol; TG, total triglycerides; HDL-C, high-density lipoprotein cholesterol; LDL-C, low-density lipoprotein cholesterol; FPG, fasting plasma glucose; HbA1c, glycated hemoglobin; FINS, fasting insulin; HOMA-IR, homeostasis model assessment of insulin resistance; 25(OH)D, serum 25-hydroxyvitamin D; PTH, serum parathyroid hormone; BMD, bone mineral density; FN, femoral neck; TH, total hip; LS, lumbar spine; P1NP, procollagen type I amino-terminal propeptide; N-MID, N-terminal osteocalcin; β-CTX, β-cross-linked C-telopeptide of type I collagen.

**P values (bold)** indicate non-normally distributed data.

**SUPPLEMENT TABLE** **2.** Bone metabolic variables of the participants at baseline and at 6 and 12 months after sleeve gastrectomy

|  |  | **Men** |  |  |  |  |  | **Women** |  |  |  |
| --- | --- | --- | --- | --- | --- | --- | --- | --- | --- | --- | --- |
|  | **baseline** | **6 months** | ***P-value*** | **12 months** | ***P-value*** |  | **baseline** | **6 months** | ***P-value*** | **12 months** | ***P-value*** |
| TH BMD, g/cm^2^ | 1.063±0.108 | 1.014±0.113 | **<0.001** | 0.972±0.099 | **<0.001** |  | 1.1249±0.130 | 1.075±0.108 | **<0.001** | 1.052±0.137 | **<0.001** |
| LS BMD, g/cm^2^ | 1.166±0.103 | 1.170±0.100 | 0.531 | 1.169±0.105 | 0.815 |  | 1.253±0.135 | 1.237±0.116 | 0.647 | 1.253±0.158 | 0.232 |
| FN BMD, g/cm^2^ | 1.039±0.112 | 1.018±0.120 | 0.015 | 0.981±0.097 | **<0.001** |  | 1.047±0.110 | 1.041±0.098 | 0.09 | 1.011±0.114 | **<0.001** |
| P1NP, mg/mL | 57.06±21.58 | 98.24±29.75 | **<0.001** | 92.87±29.12 | **<0.001** |  | 49.08(37.16, 69.56) | 57.19(50.9, 69.22) | 0.06 | 63.45(47.08, 78.31) | **0.03** |
| N-MID, mg/mL | 15.50±4.60 | 32.43±11.00 | **<0.001** | 34.06±11.92 | **<0.001** |  | 11.98(9.60, 17.26) | 19.46(18.34,22.75) | **0.001** | 20.81±7.72 | **<0.001** |
| β-CTX, mg/mL | 463.09±181.59 | 1211.60±352.75 | **<0.001** | 991.20±363.76 | **<0.001** |  | 267.10(182.28, 372.05) | 769.07±255.61 | **0.002** | 635.61±265.11 | **<0.001** |

Data are presented as n (%), mean ± standard deviation, or median (Q25 and Q75).

***P* values (bold)** indicate statistical significance compared with baseline in each sex group.

**Abbreviations:** BMD, bone mineral density; FN, femoral neck; TH, total hip; LS, lumbar spine; P1NP, procollagen type I amino-terminal propeptide; N-MID, N-terminal osteocalcin; β-CTX, β-cross-linked C-telopeptide of type I collagen.
